# Quantum-squeezing effects of strained multilayer graphene NEMS

**DOI:** 10.1186/1556-276X-6-355

**Published:** 2011-04-20

**Authors:** Yang Xu, Sheping Yan, Zhonghe Jin, Yuelin Wang

**Affiliations:** 1 Department of Information Science and Electronic Engineering, Zhejiang University, Hangzhou 310027, China; 2 State Key Laboratory of Transducer Technology, Shanghai Institute of Metallurgy Chinese Academy of Sciences, Shanghai 100050, China

## Abstract

Quantum squeezing can improve the ultimate measurement precision by squeezing one desired fluctuation of the two physical quantities in Heisenberg relation. We propose a scheme to obtain squeezed states through graphene nanoelectromechanical system (NEMS) taking advantage of their thin thickness in principle. Two key criteria of achieving squeezing states, zero-point displacement uncertainty and squeezing factor of strained multilayer graphene NEMS, are studied. Our research promotes the measured precision limit of graphene-based nano-transducers by reducing quantum noises through squeezed states.

## Introduction

The Heisenberg uncertainty principle, or the standard quantum limit [1,2], imposes an intrinsic limitation on the ultimate sensitivity of quantum measurement systems, such as atomic forces [3], infinitesimal displacement [4], and gravitational-wave [5] detections. When detecting very weak physical quantities, the mechanical motion of a nano-resonator or nanoelectromechanical system (NEMS) is comparable to the intrinsic fluctuations of the systems, including thermal and quantum fluctuations. Thermal fluctuation can be reduced by decreasing the temperature to a few mK, while quantum fluctuation, the quantum limit determined by Heisenberg relation, is not directly dependent on the temperature. Quantum squeezing is an efficient way to decrease the system quantum [6-8]. Thermomechanical noise squeezing has been studied by Rugar and Grutter [9], where the resonator motion in the fundamental mode was parametrically squeezed in one quadrature by periodically modulating the effective spring constant at twice its resonance frequency. Subsequently, Suh et al. [10] have successfully achieved parametric amplification and back-action noise squeezing using a qubit-coupled nanoresonator.

To study quantum-squeezing effects in mechanical systems, zero-point displacement uncertainty, Δ*x*_zp_, the best achievable measurement precision, is introduced. In classical mechanics, the complex amplitudes, *X *= *X*_1 _+ i*X*_2_, where *X*_1 _and *X*_2 _are the real and imaginary parts of complex amplitudes respectively, can be obtained with complete precision. In quantum mechanics, *X*_1 _and *X*_2 _do not commute, with the commutator [*X*_1_, *X*_2_] = i*ħ*/*M*_eff_*w*, and satisfy the uncertainty relationship Δ*X*_1_Δ*X*_2 _≥ (*ħ*/2*M*_eff_*w*)^1/2^. Here, *ħ *is the Planck constant divided by 2π, *M*_eff _= 0.375*ρLWh*/2 is the effective motional double-clamped film mass [11,12], *ρ *is the volumetric mass density, *L*, *W*, and *h *are the length, width, and thickness of the film, respectively, and *w *= 2*f*_0 _is the fundamental flexural mode angular frequency with(1)

where *E *is the Young's modulus of the material, *T*_s _is the tension on the film, *A *is 0.162 for a cantilever and *A *is 1.03 for a double-clamped film [13]. Therefore, Δ*x*_zp _of the fundamental mode of a NEMS device with a double-clamped film can be given by Δ*x*_zp _= Δ*X*_1 _= Δ*X*_2 _= (*ħ*/2*M*_eff_*w*)^1/2^. In a mechanical system, quantum squeezing can reduce the displacement uncertainty Δ*x*_zp_.

Recently, free-standing graphene membranes have been fabricated [14], providing an excellent platform to study quantum-squeezing effects in mechanical systems. Meanwhile, a graphene membrane is sensitive to external influences, such as atomic forces or infinitesimal mass (e.g., 10^-21 ^g) due to its atomic thickness. Although graphene films can be used to detect very infinitesimal physical quantities, the quantum fluctuation noise Δ*x*_zp _of graphene NEMS devices (approx. 10^-2 ^nm), could easily surpass the magnitudes of signals caused by external influences. Thus, quantum squeezing becomes necessary to improve the ultimate precision of graphene-based transducers with ultra-high sensitivity. In this study, we have studied quantum-squeezing effects of strained multilayer graphene NEMS based on experimental devices proposed by Chen et al. [15].

## Results

### Displacement uncertainty of graphene NEMS

**Figure 1:**
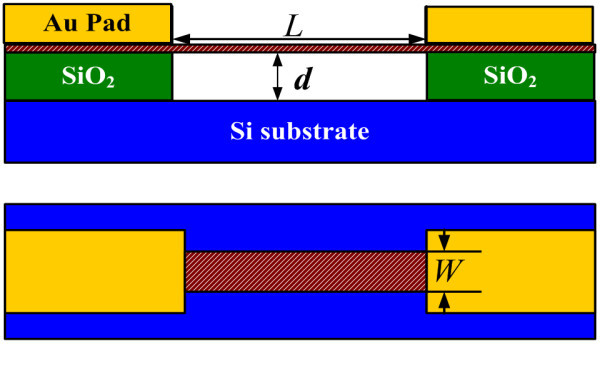
**Schematic of a double-clamped graphene NEMS device**.

A typical NEMS device with a double-clamped free-standing graphene membrane is schematically shown in Figure 1. The substrate is doped Si with high conductivity, and the middle layer is SiO_2 _insulator. A pump voltage can be applied between the membrane and the substrate. The experimental data of the devices are used in our simulation [15]. For graphene, we use a Young's modulus of *E *= 1.03 × 10^12 ^Pa, volumetric mass density of *ρ *= 2200 kg/m^3^, based on previous theories and scanning tunneling microscope experiments [13,15,16].

**Figure 2:**
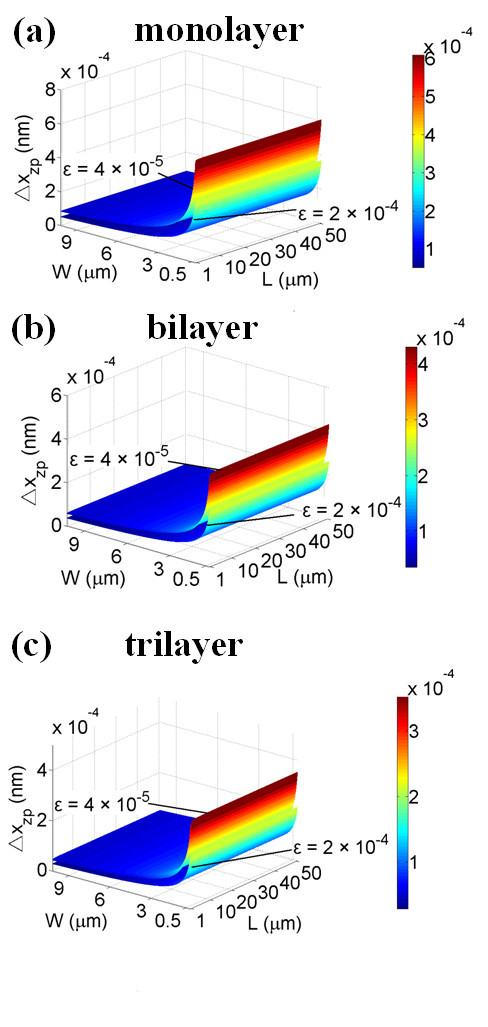
**Δ*x***_**zp **_**versus multilayer graphene film sizes with strains**. **(a) **Monolayer graphene. **(b) **Bilayer graphene. **(c) **Trilayer graphene.

In graphene sensors and transducers, to detect the molecular adsorbates or electrostatic forces, a strain *ε *will be generated in the graphene film [15,17]. When a strain exists in a graphene film, the tension *T*_s _in Equation 1 can be deduced as *T*_s _= *ESε *= *EWhε*. The zero-point displacement uncertainty of the strained graphene film is given by(2)

where *ρ*' represents the effective volumetric mass density of graphene film after applying strain. The typical measured strains in [15] are *ε *= 4 × 10^-5 ^when *ρ*' = 4*ρ *and *ε *= 2 × 10^-4 ^when *ρ*' = 6*ρ*. Based on Equation 2, measurable Δ*x*_zp _of the strained multilayer graphene films of various sizes are shown in Figure 2, and typical Δ*x*_zp _values of graphene NEMS under various *ε *are summarized in Table 1.

**Table 1:** Calculated Δ*x*_zp _(10^-4^nm) of monolayer (Mon), bilayer (Bi), and trilayer (Tri) graphene versus strain *ε *(*L *= 1.1 μm, *W *= 0.2 μm)

*ε *= 0			***ε *= 4 × 10**^**-5**^			***ε *= 2 × 10**^**-4**^		
**Mon**	**Bi**	**Tri**	**Mon**	**Bi**	**Tri**	**Mon**	**Bi**	**Tri**
**34.0**	**17.0**	**11.3**	**6.05**	**4.23**	**3.39**	**3.67**	**2.59**	**2.10**

According to the results in Figure 2 and Table 1, we find Δ*x*_zp_^large strain ^< Δ*x*_zp_^small strain^; one possible reason is that larger applied strain results in smaller fundamental angular frequency and Δ*x*_zp_, therefore, the quantum noise can be reduced.

### Quantum-squeezing effects of graphene NEMS

To analyze quantum-squeezing effects in graphene NEMS devices, a back-action-evading circuit model is used to suppress the direct electrostatic force acting on the film and modulate the effective spring constant *k *of the membrane film. Two assumptions are used, namely, the film width *W *is on the micrometer scale and *X*_1 _>>*d*, where *d *is the distance between the film and the substrate. Applying a pump voltage *V*_m_(*t*) = *V*[1*+*sin(2*w*_m_*t *+ *θ*)], between the membrane film and the substrate, the spring constant *k *will have a sinusoidal modulation *k*_m_(*t*), which is given by *k*_m_(*t*) = sin(2*w*_m_*t *+ *θ*)*C*_T_*V*^2^/2*d*^2^, where *C*_T _is the total capacitance composed of structure capacitance *C*_0_, quantum capacitance *C*_q_, and screen capacitance *C*_s _in series [18]. The quantum capacitance *C*_q _and screen capacitance *C*_s _cannot be neglected [18-20] owing to a graphene film thickness on the atomic scale. The quantum capacitance of monolayer graphene [21,22] is *C*_q_^monolayer ^= 2e^2^*n*^1/2^/(*ħv*_F_π^1/2^), where *n *is the carrier concentration, *e *is the elementary charge, and *v*_F _≈ *c*/300, where *c *is the velocity of light, with bilayer *C*_q_^bilayer ^= 2 × 0.037*m*_e_*e*^2^/π*ħ*^2^, and trilayer *C*_q_^trilayer ^= 2 × 0.052*m*_e_*e*^2^/π*ħ*^2^, where *m*_e _is the electron mass [23].

**Figure 3:**
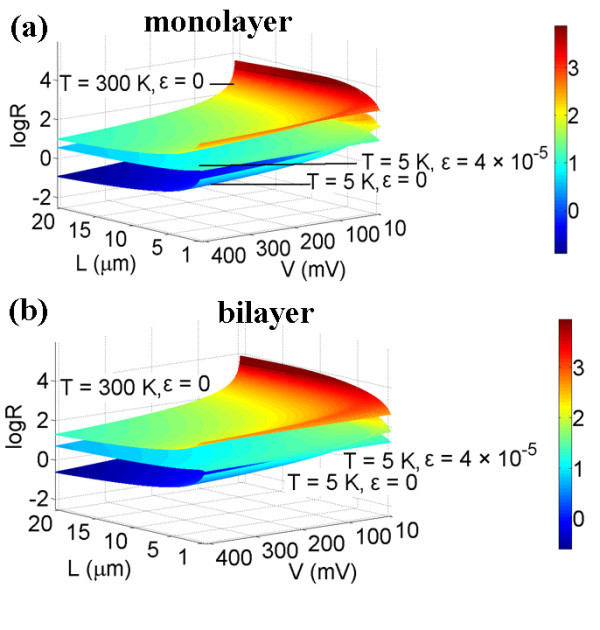
**Log *R *versus applied voltages for graphene film structures at *T *= 300 K with *Q *= 125 and *T *= 5 K with *Q *= 14000**. **(a) **Monolayer graphene and **(b) **bilayer graphene.

**Figure 4:**
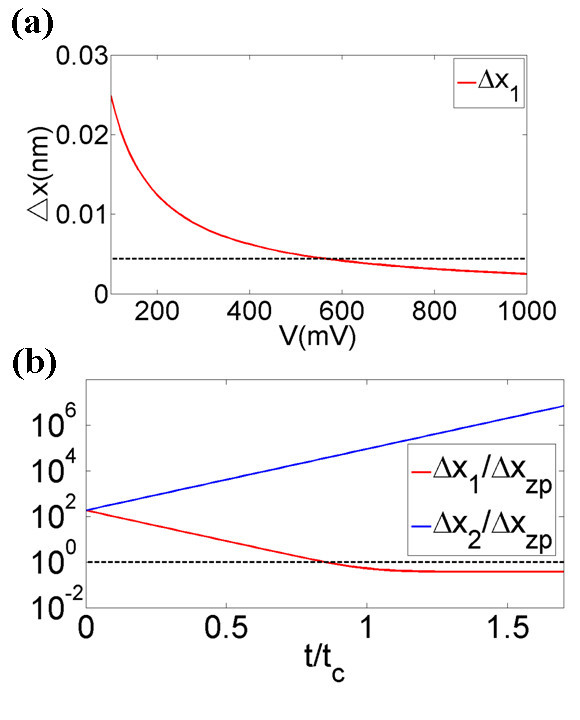
**(a) Δ*X*_1 _versus applied voltages of graphene film and the dashed reference line is Δ*X *= Δ*x*_zp_**. **(b) **Time dependences of Δ*X*_1 _and Δ*X*_2_, which are expressed in units of Δ*x*_zp_, where time is in units of *t*_ct_, *θ *= 0, and the dashed reference line is Δ*X *= Δ*x*_zp_. *L *= 1.1 μm, *W *= 0.2 μm, *d *= 0.1 μm, *T *= 5 K, *Q *= 14000, and *V *= 2.5V.

Pumping the graphene membrane film from an initial thermal equilibrium state at frequency *w*_m _= *w*, the variance of the complex amplitudes, Δ*X*^2^_1,2_(*t*, *θ*), are given by [24](3)

where * N *= [exp(*ħw/k*_B_*T*) - 1]^-1 ^is the average number of quanta at absolute temperature *T *and frequency *w*, *k*_B _is the Boltzmann constant, *τ *= *Q*/*w *is the relaxation time of the mechanical vibration, *Q *is the quality factor of the NEMS, and *η *= *C*_T_*V*^2^/8*d*^2^*M*_eff_*w*_m_. When *θ *= 0, a maximum modulation state, namely, the best quantum-squeezed state, can be reached [9,21], and Δ*X*_1 _can be simplified as Δ*X*_1_(*t*) = [(*ħ*/2*M*_eff_*w*_a_)(2*N *+ 1)(τ^-1 ^+ 2*η*)^-1^(τ^-1 ^+ 2*η*exp(-τ^-1 ^+ 2*η*)*t*)]^1/2^. As *t *→ ∞, the maximum squeezing of Δ*X*_1 _is always finite, with expression of Δ*X*_1_(*t *→ ∞) ≈ [*ħ*(2*N *+ 1)(1 + 2*Qη*)^-1^/2*M*_eff_*w*]^1/2^. The squeezing factor *R*, defined as *R *= Δ*X*_1_/Δ*x*_zp _= Δ*X*_1_/(*ħ*/2*M*_eff_*w*)^1/2^, can be expressed as(4)

where *ε *is the strain applied on the graphene film. In order to achieve quantum squeezing, *R *must be less than 1. According to Equation 4, *R *values of monolayer and bilayer graphene films with various dimensions, strain *ε*, and applied voltages at *T *= 300 K and *T *= 5 K have been shown in Figure 3. Quantum squeezing is achievable in the region log *R *< 0 at *T *= 5 K. As shown in Figure 3, the applied strain increases the *R *values because of the increased fundamental angular frequency and the decreased Δ*x*_zp _caused by strain, which makes squeezing conditions more difficult to reach. Figure 4a has shown that Δ*X*_1 _changes with applied voltages at *T *= 5 K, the red line represents the uncertainties of *X*_1 _and the dashed reference line is Δ*X *= Δ*x*_zp_. As shown in Figure 4a, applying a voltage larger than 100 mV, we can obtain Δ*X*_1 _< Δ*x*_zp_, which means that the displacement uncertainty is squeezed, and the quantum squeezing is achieved. Some typical *R *values of monolayer graphene film, obtained by varying the applied voltage *V*, such as strain *ε*, have been listed in Table 2 (with *T *= 300 K and *Q *= 125) and Table 3 (with *T *= 5 K and *Q *= 14000). As shown in Tables 2 and 3 and Figure 3, lowering the temperature to 5 K can dramatically decrease the *R *values. The lower the temperature, the larger the quality factor *Q*, which makes the squeezing effects stronger.

**Table 2:** *R *values of monolayer graphene versus various strain *ε *and voltage *V *(*L *= 1.1 μm, *W *= 0.2 μm, and *T *= 300 K with *Q *= 125)

	*ε *= 0	***ε *= 4 × 10**^**-5**^	***ε *= 2 × 10**^**-4**^
***V *= 2 V**	**38.33**	**198.15**	**259.14**
***V *= 10 V**	**7.669**	**42.84**	**69.86**

**Table 3:** *R *values of monolayer graphene versus various strain *ε *and voltage *V *(*L *= 1.1 μm, *W *= 0.2 μm, and *T *= 5 K with *Q *= 14000)

	*ε *= 0	***ε *= 4 × 10**^**-5**^	***ε *= 2 × 10**^**-4**^
***V *= 2 V**	**0.468**	**2.620**	**4.319**
***V *= 10 V**	**0.0936**	**0.524**	**0.867**

In contrast to the previous squeezing analysis proposed by Rugar and Grutter [9], in which steady-state solutions have been assumed and the minimum *R *is 1/2, we use time-dependent pumping techniques to prevent *X*_2 _from growing without bound as *t *→ ∞, which should be terminated after the characteristic time *t*_ct _= ln(*QC*_T_*V*^2^/4*M*_eff_*w*^2^*d*^2^)4*M*_eff_*wd*^2^/*C*_T_*V*^2^, when *R *achieves its limiting value. Therefore, we have no upper bound on *R*. Figure 4b has shown the time dependence of Δ*X*_1 _and Δ*X*_2 _in units of *t*_ct_, and the quantum squeezing of the monolayer graphene NEMS has reached the limiting value after one *t*_ct _time. Also, to make the required heat of conversion from mechanical energy negligible during the pump stage, *t*_ct _<<*τ *must be satisfied. We find *t*_ct_/*τ *≈ 1.45 × 10^-5 ^for the monolayer graphene parameters considered in the text.

## Discussion

**Figure 5:**
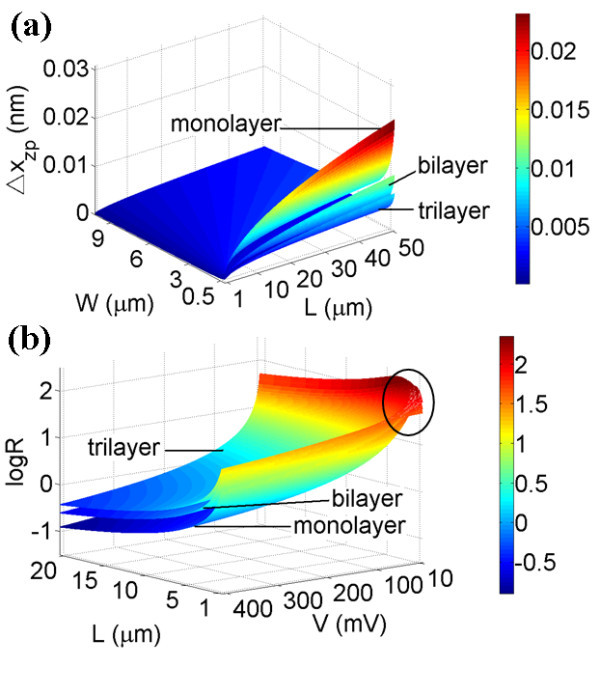
**(a) Δ*x*_zp _versus various graphene film sizes**. **(b) **Log *R *versus multilayer graphene film lengths and applied voltages at *T *= 5 K

The ordering relation of Δ*x*_zp _for multilayer graphene is Δ*x*_zp_^trilayer ^< Δ*x*_zp_^bilayer ^< Δ*x*_zp_^monolayer ^shown in Figure 5a, as the zero-point displacement uncertainty is inversely proportional to the film thickness. Squeezing factors *R *of multilayer graphene films follow the ordering relation; *R*_trilayer _>*R*_bilayer _>*R*_monolayer_, as shown in Figure 5b, as *R *is proportional to the thickness of the graphene film. The thicker the film, the more difficult it is to achieve a quantum-squeezed state, which also explains why traditional NEMS could not achieve quantum squeezing due to their thickness of several hundred nanometers.

**Figure 6:**
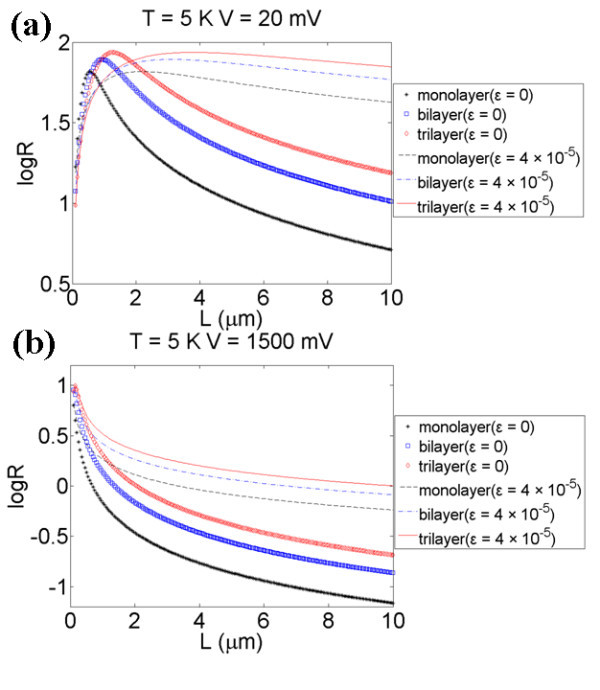
***R *versus *L *with *ε *= 0.4 × 10**^**-5**^**, and *V *= 20 mV, 1.5 V**.

For a clear view of squeezing factor *R *as a function of film length *L*, 2D curves from Figure 5b are presented in Figure 6. It is found that *R *approaches unity as *L *approaches zero, while *R *tends to be zero as *L *approaches infinity as shown in Figure 6a,b. It explains why *R *has some kinked regions, shown in the upper right part of Figure 5b with black circle, when the graphene film length is on the nanometer scale shown in Figure 3. To realize quantum squeezing, the graphene film length should be in the order of a few micrometers and the applied voltage *V *should not be as small as several mV, shown in Figure 6b. As *L *→ 0, where the graphene film can be modeled as a quantum dot, the voltage must be as large as a few volts to modulate the film to achieve quantum squeezing. As *L *→ ∞, where graphene films can be modeled as a 1D chain, the displacement uncertainty would be on the nanometer scale so that even a few mV of pumping voltage can modulate the film to achieve quantum squeezing easily.

By choosing the dimensions of a typical monolayer graphene NEMS device in [15] with *L *= 1.1 μm, *W *= 0.2 μm, *T *= 5 K, *Q *= 14000, *V *= 2.5 V, and *ε *= 0, we obtain Δ*x*_zp _= 0.0034 nm and *R *= 0.374. After considering quantum squeezing effects based on our simulation, Δ*x*_zp _can be reduced to 0.0013 nm. With a length of 20 μm, Δ*x*_zp _can be as large as 0.0145 nm, a radio-frequency single-electron-transistor detection system can in principle attain such sensitivities [25]. In order to verify the quantum squeezing effects, a displacement detection scheme need be developed.

## Conclusions

In conclusion, we presented systematic studies of zero-point displacement uncertainty and quantum squeezing effects in strained multilayer graphene NEMS as a function of the film dimensions *L*, *W*, *h*, temperature *T*, applied voltage *V*, and strain *ε *applied on the film. We found that zero-point displacement uncertainty Δ*x*_zp _of strained graphene NEMS is inversely proportional to the thickness of graphene and the strain applied on graphene. By considering quantum capacitance, a series of squeezing factor *R *values have been obtained based on the model, with *R*_monolayer _<*R*_bilayer _<*R*_trilayer _and *R*_small strain _<*R*_large strain _being found. Furthermore, high-sensitivity graphene-based nano-transducers can be developed based on quantum squeezing.

## Abbreviation

NEMS, nanoelectromechanical system.

## Competing interests

The authors declare that they have no competing interests.

## Authors' contributions

Both SY and YX designed and conducted all the works and drafted the manuscript. Both ZJ and YW have read and approved the final manuscript.

## References

[B1] LaHaye MD Buu O Camarota B Schwab KC Approaching the quantum limit of a nanomechanical resonator Science 2004 304 74 77 10.1126/science.1094419 15064412

[B2] Blencowe M Nanomechanical quantum limits Science 2004 304 56 57 10.1126/science.1095768 15060311

[B3] Caves CM Thorne KS Drever RWP Sandberg VD Zimmermann M ON the measurement of a weak classical force coulped to a quantum-mechanical oscillator. I. Issues of principle Rev Mod Phys 1980 52 341 392 10.1103/RevModPhys.52.341

[B4] Mozyrsky D Martin I Hastings MB Quantum-limited sensitivity of single-electron-transistor-based displacement detectors Phys Rev Lett 2004 92 083103 10.1103/PhysRevLett.92.018303 14754026

[B5] Hollenhorst JN Quantum limits on resonant-mass gravitational-radiation detectors Phys Rev D 1979 19 1669 1679 10.1103/PhysRevD.19.1669

[B6] Blencowe M Quantum electromechanical systems Phys Rep Rev Sec Phys Lett 2004 395 159 222

[B7] Giovannetti V Lloyd S Maccone L Quantum-enhanced measurements: beating the standard quantum limit Science 2004 306 1330 1336 10.1126/science.1104149 15550661

[B8] Blencowe MP Wybourne MN Quantum squeezing of mechanical motion for micron-sized cantilevers Physica B 2000 280 555 556 10.1016/S0921-4526(99)01862-1

[B9] Rugar D Grutter P Mechanical parametric amplification and thermomechanical noise squeezing Phys Rev Lett 1991 67 699 702 10.1103/PhysRevLett.67.699 10044966

[B10] Suh J LaHaye MD Echternach PM Schwab KC Roukes ML Parametric amplification and back-action noise squeezing by a qubit-coupled nanoresonator Nano Lett 2010 10 3990 3994 10.1021/nl101844r 20843059

[B11] Ekinci KL Yang YT Roukes ML Ultimate limits to inertial mass sensing based upon nanoelectromechanical systems J Appl Phys 2004 95 2682 2689 10.1063/1.1642738

[B12] Ekinci KL Roukes ML Nanoelectromechanical systems Rev Sci Instrum 2005 76 061101 10.1063/1.1927327

[B13] Bunch JS van der Zande AM Verbridge SS Frank IW Tanenbaum DM Parpia JM Craighead HG McEuen PL Electromechanical resonators from graphene sheets Science 2007 315 490 493 10.1126/science.1136836 17255506

[B14] Novoselov KS Geim AK Morozov SV Jiang D Zhang Y Dubonos SV Grigorieva IV Firsov AA Electric field effect in atomically thin carbon films Science 2004 306 666 669 10.1126/science.1102896 15499015

[B15] Chen CY Rosenblatt S Bolotin KI Kalb W Kim P Kymissis I Stormer HL Heinz TF Hone J Performance of monolayer graphene nanomechanical resonators with electrical readout Nat Nanotechnol 2009 4 861 867 10.1038/nnano.2009.267 19893525

[B16] Ni ZH Wang HM Kasim J Fan HM Yu T Wu YH Feng YP Shen ZX Graphene thickness determination using reflection and contrast spectroscopy Nano Lett 2007 7 2758 2763 10.1021/nl071254m 17655269

[B17] Lee C Wei XD Kysar JW Hone J Measurement of the elastic properties and intrinsic strength of monolayer graphene Science 2008 321 385 388 10.1126/science.1157996 18635798

[B18] Xu Y Aluru NR Pull-in/out analysis of nano/microelectromechanical switches with defective oxide layers Appl Phys Lett 2009 95 073112 10.1063/1.3211111

[B19] Tang Z Xu Y Li G Aluru NR Physical models for coupled electromechanical analysis of silicon nanoelectromechanical systems J Appl Phys 2005 97 114304 10.1063/1.1897483

[B20] Xu Y Aluru NR Multiscale electrostatic analysis of silicon nanoelectromechanical systems (NEMS) via heterogeneous quantum models Phys Rev B 2008 77 075313

[B21] Fang T Konar A Xing HL Jena D Carrier statistics and quantum capacitance of graphene sheets and ribbons Appl Phys Lett 2007 91 092109 10.1063/1.2776887

[B22] Xia JL Chen F Li JH Tao NJ Measurement of the quantum capacitance of graphene Nat Nanotechnol 2009 4 505 509 10.1038/nnano.2009.177 19662012

[B23] Koshino M Ando T Orbital diamagnetism in multilayer graphenes: systematic study with the effective mass approximation Phys Rev B 2007 76 085425

[B24] Grishchuk LP Sazhin MV Squeezed quantum states of a harmonic-oscillator in the problem of detecting gravitational-waves Zh Eksp Teor Fiz 1983 84 1937 1950

[B25] Turin VO Korotkov AN Analysis of the radio-frequency single-electron transistor with large quality factor Appl Phys Lett 2003 83 2898 2900 10.1063/1.1614840

